# The Moxibustion-Induced Thermal Transport Effect Between the Heart and Lung Meridians With Infrared Thermography

**DOI:** 10.3389/fcvm.2022.817901

**Published:** 2022-05-13

**Authors:** Xiaoyu Li, Yongliang Jiang, Hantong Hu, Jiali Lou, Yajun Zhang, Xiaofen He, Yuanyuan Wu, Junfan Fang, Xiaomei Shao, Jianqiao Fang

**Affiliations:** ^1^Key Laboratory of Acupuncture and Neurology of Zhejiang Province, Department of Neurobiology and Acupuncture Research, The Third Clinical Medical College, Zhejiang Chinese Medical University, Hangzhou, China; ^2^The Third Affiliated Hospital of Zhejiang Chinese Medical University, Hangzhou, China

**Keywords:** heart and lung meridians, site specificity, infrared thermography, moxibustion, thermal transport

## Abstract

**Objectives:**

By comparing the differences in the thermal transport effect between the heart and lung meridians induced by moxibustion, this study aimed to investigate the specificity of site-to-site associations on the body surface between different meridians.

**Methods:**

Eighty healthy participants were divided into the heart meridian intervention group and the lung meridian intervention group; moxibustion was performed at these two meridians, respectively. Baseline temperature and its change magnitude from baseline induced by moxibustion in 6 measuring sites of the heart and lung meridians were assessed by infrared thermography (IRT). Measuring sites included: Site 1 (Chize, LU5), Site 2 (midpoint of LU9 and LU5), Site 3 (Taiyuan, LU9), Site 4 (Shaohai, HT3), Site 5 (midpoint of HT7 and HT3), and Site 6 (Shenmen, HT7).

**Results:**

Forty participants (20 male and 20 female, 27.90 ± 0.52 years) were assigned to the heart meridian intervention group, and 40 participants (20 male and 20 female, 28.08 ± 0.54 years) were assigned to the lung meridian intervention group. In the lung meridian intervention group (moxibustion over LU5), the temperature of the distal sites in the lung meridian increased significantly at 5, 10, and 15 min compared with pre-moxibustion (*P* < 0.001). The temperature of Site 4 in the heart meridian, which was nearest to the moxibustion site, increased significantly compared with pre-moxibustion (*P* < 0.05), while the temperature in the distal sites of the heart meridian did not differ significantly during moxibustion. Regarding the comparison of temperature change magnitude from baseline (ΔT) between the two meridians, the ΔT of Site 2 in the lung meridian was significantly higher than Site 4 in the heart meridian at 5 and 10 min after moxibustion (*P* < 0.05), despite that Site 2 was more distal from the moxibustion site than Site 4. Similarly, the ΔT of Site 3 in the lung meridian was significantly higher than Site 5 and Site 6 in the heart meridian at 5, 10, and 15 min after moxibustion (*P* < 0.05). In the heart meridian invervention group, similar thermal transport effect between the two meridians was observed. The thermal transport effect of the distal sites along the heart meridian was more significant than that of the site closer to the moxibustion site but located in the lung meridian. Taken together, aforementioned results indicated that the moxibustion-induced thermal transport effect between the heart and lung meridians is generally more significant in the distal sites along the corresponding meridian than that in the closer sites of the other meridian.

**Conclusions:**

In the heart and lung meridians, the moxibustion-induced thermal transport effect is closely related to meridian routes, not just related to the absolute distance from the moxibustion site, thereby confirming the relative specificity of “site-to-site” associations on the body surface in these two meridians.

**Systematic Review Registration:**

https://clinicaltrials.gov/ct2/show/NCT05330403, identifier NCT05330403.

## Introduction

Evidence-based medicine suggests that acupuncture is effective for treating a wide range of diseases (1–3), such as migraine, cancer pain, constipation, and other diseases (4–6). Therefore, acupuncture is becoming internationally recognized in recent years.

The meridian theory, which is the theoretical basis of acupuncture therapy, plays an extremely important role in guiding clinical acupuncture practice, especially in the selection of acupuncture points. According to the meridian theory, meridians and acupuncture points are considered to be both sensitive areas for disease response and acupuncture stimulation points. Although systematic research on meridians dates back to as early as the 1950s, no major breakthroughs have been made yet, and the essence of meridians remains unsolved. Moreover, there are many limitations in the methodologies of previous meridian studies (7–9). These limitations lead doubts regarding the validity of the meridian theory and the physical existence of meridians. Thus, researchers has been seeking help from modern technology. Multiple modern techniques have been adopted in meridian research, such as laser doppler flowmetry (10–12), infrared thermography (IRT) (13, 14) and near-infrared spectroscopy (15, 16). Among these techniques, IRT can dynamically detect the temperature of the body surface with the advantages of high sensitivity, good visualization, and noninvasiveness, so it has been widely used in traditional Chinese medicine and disease diagnosis, as well as meridian studies (17–19). However, the majority of previous meridian studies involving IRT aimed to explore the correlation between one meridian and its corresponding organ (20, 21). To the best of our knowledge, there are no existing studies that have been designed to explore the specificity of site-to-site associations on the body surface between different meridians by using IRT.

The specificity of site-to-site associations on the body surface between different meridians is an extremely important topic in meridian studies, especially for explaining the connection between meridian distribution routes and meridian treatment scopes. In details, the heart meridian and the lung meridian both have a roughly parallel distribution pattern on the surface of the body, with their distribution routes on the inner side of the arm. Although the treatment scope of the lung meridian and the heart meridian are not exactly the same, they are firmly interconnected. For example, electroacupuncture on both the heart meridian and the lung meridian can improve myocardial ischemia, but the effect of electroacupuncture on the heart meridian is relatively specific and superior (22). This shows that meridians have relative specificity of “site-to-site” associations on the body surface, but explicit rules and patterns of such associations need further investigation.

Meanwhile, previous studies have demonstrated that the temperature change in distal sites (i.e., thermal transport effect) induced by acupuncture and moxibustion seems to be meridian-specific. Human meridians appear to be favorable channels for thermal transport (19, 23, 24). Thus, the thermal transport characteristic is one of the unique characteristic of meridians.

Therefore, the aim of this study was to explore the specificity of site-to-site association between different meridians by comparing moxibustion-induced thermal transport responses with IRT. In this study, the heart and lung meridians were chosen as the two meridians for comparison. Moxibustion was administered to either the heart meridian or the lung meridian in healthy subjects, and IRT was used to record thermographs of these two meridians in the forearm before, during and after moxibustion.

## Materials and Methods

### Study Design

**Figure 1:**
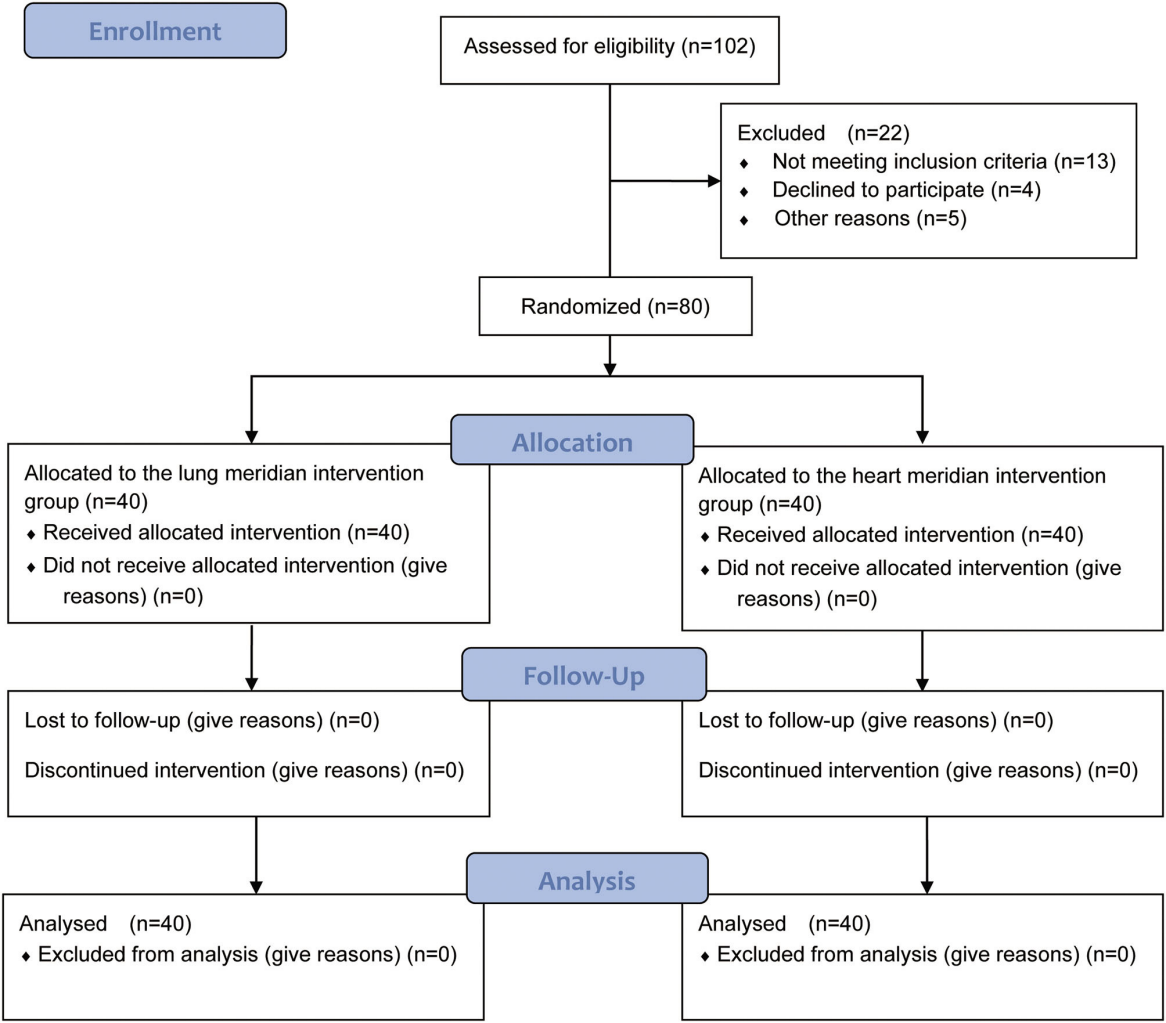
Flow diagram of the participants through the study [With reference to the CONSORT 2010 Flow Diagram, Schulz et al., BMJ, 2010, 340: p. c332; (9)].

This clinical trial was conducted in the Third Affiliated Hospital of Zhejiang Chinese Medical University. Eighty participants were divided into the heart meridian moxibustion group and the lung meridian moxibustion group. The study protocol has been registered in the Clinicaltrials registry with the identification code NCT05330403. Figure 1 shows the flow diagram of the trial.

### Sample Size Estimation

This trial was a meridian study in which IRT was used to assess the baseline temperature and moxibustion-induced temperature change in two meridians. No existing references could be used to calculate the required sample size. Thus, the required sample size was mainly estimated on the basis of the feasibility and the funds of our trial. Finally, 80 participants were included, with 40 subjects in each group.

### Participants

Inclusion criteria for the healthy volunteers: (1) had recent medical examination records confirming the absence of cardiovascular, respiratory, digestive, urinary, hematological, endocrine and neurological diseases; (2) 20 ≤ age ≤ 40 years, male or female; (3) had the ability to communicate with others normally; (4) an understanding of the entire study protocol; (5) signed written informed consent. The exclusion criteria of the healthy volunteers were as follows: (1) had mental illnesses, severe depression, alcohol dependence or a history of drug abuse; (2) pregnant or lactating; (3) were participating in other trials.

The study processes were approved by the Ethics Committee of the Third Affiliated Hospital of Zhejiang Chinese Medical University (approval no.: ZSLL-KY-2019-001G-01). All participants signed informed consent forms.

### Randomization and Allocation

SAS 9.3.1 software (SAS Institute Inc., Cary, NC, USA) was used to generate a randomized grouping table. To ensure the same gender distribution in both groups, randomization is stratified by sex. Envelopes for the randomized grouping table were opaque, numbered in order, and contain the randomized serial numbers for grouping. According to the randomized serial numbers, all included participants were successfully randomized to the lung meridian intervention group or the heart meridian intervention group in a 1:1 ratio.

### Blinding

Given to the unique characteristics of moxibustion, it was impractical to blind operators of moxibustion. Nevertheless, IRT examination and statistical analysis were performed by different researchers independently. Outcome assessment was performed by assessors blinded to the allocation information. Statistical analyses were performed by statisticians blinded to the allocation information.

### Examination Environment and Equipment

An experimental room was set up, and the temperature for the examination environment remained within 25-26. The relative humidity was controlled between 30 and 40%. There was no direct sunlight or obvious air convection in the experimental room.

A thermograph (NEC InfRec R450, Avio Infrared Technologies Co., Ltd., Tokyo) was used to record thermal images. Thermal images were analyzed by the configured software NS9500Std. The spectrum range of the device was 8–14 μm, the measurement range was −40-500°C, the spatial resolution was 0.58 mrad, the temperature measurement accuracy was ± 1°C, and the focusing range was 0.1–10 m. The calibrated infrared camera was fixed on a tripod 1 m away from the subject to ensure that no shaking movements or vibrations occurred during the recording of thermal images.

### Procedures for the IRT Examination and Moxibustion

All subjects were asked not to consume tea, alcohol, caffeine, or other excitable substances, not to smoke within 24 h before the experiment and not to take any drugs that affect vascular activity within 1 month. They were also informed to remain silent, breathe normally and avoid limb movements during the entire IRT recording period.

**Figure 2:**
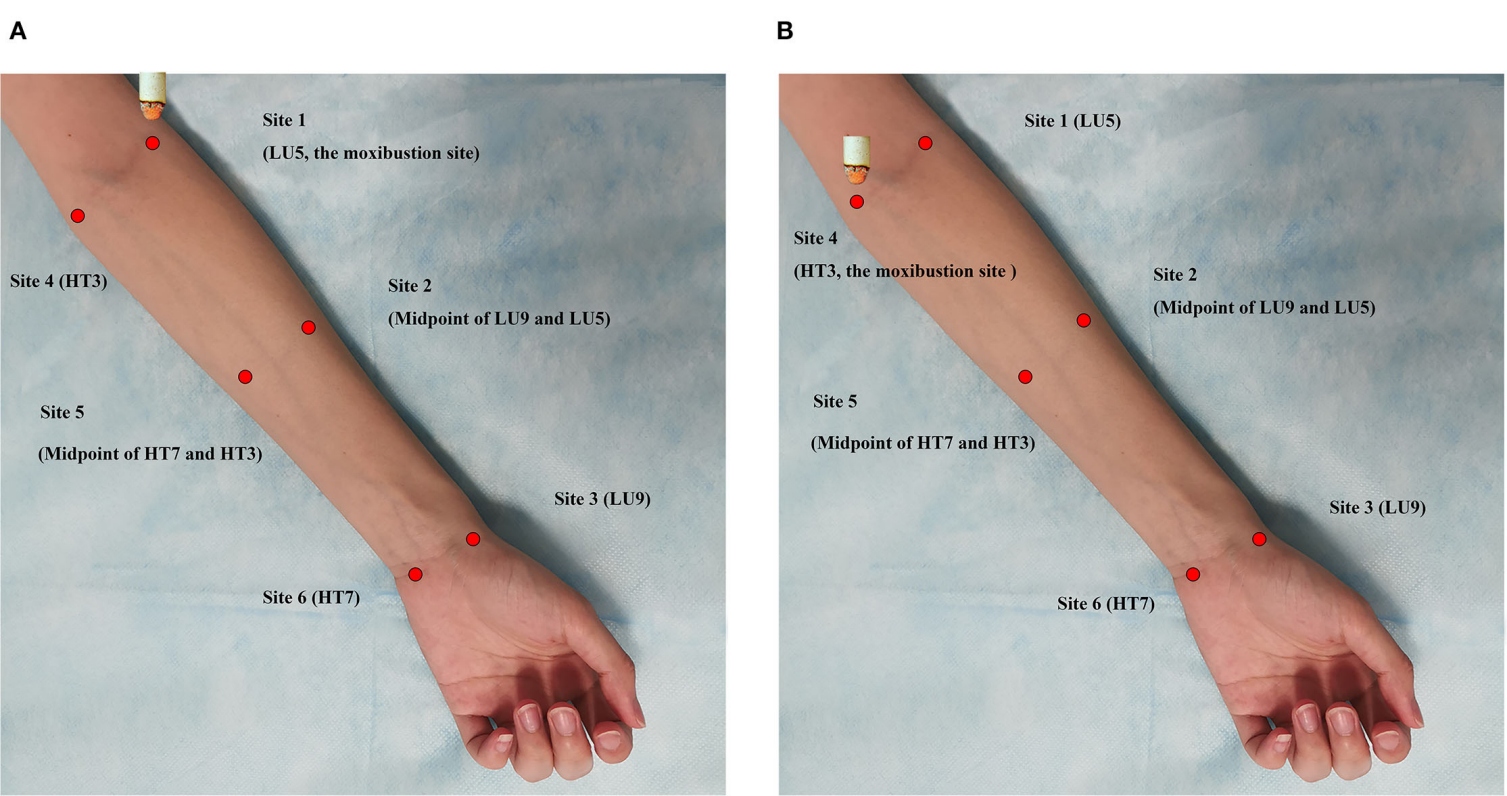
The schematic of the experiment and the positions of monitoring points. **(A)** The Lung meridian intervention group. Moxibusition site: Site 1 (LU5); IRT monitoring sites: Site 1 (LU5), Site 2 (Midpoint of LU9 and LU5), Site 3 (LU9), Site 4 (HT3), Site 5 (Midpoint of HT7 and HT3), Site 6 (HT7). **(B)** The Heart meridian intervention group. Moxibusition site: Site 4 (HT3); IRT monitoring sites: Site 1 (LU5), Site 2 (Midpoint of LU9 and LU5), Site 3 (LU9), Site 4 (HT3), Site 5 (Midpoint of HT7 and HT3), Site 6 (HT7).

**Figure 3:**
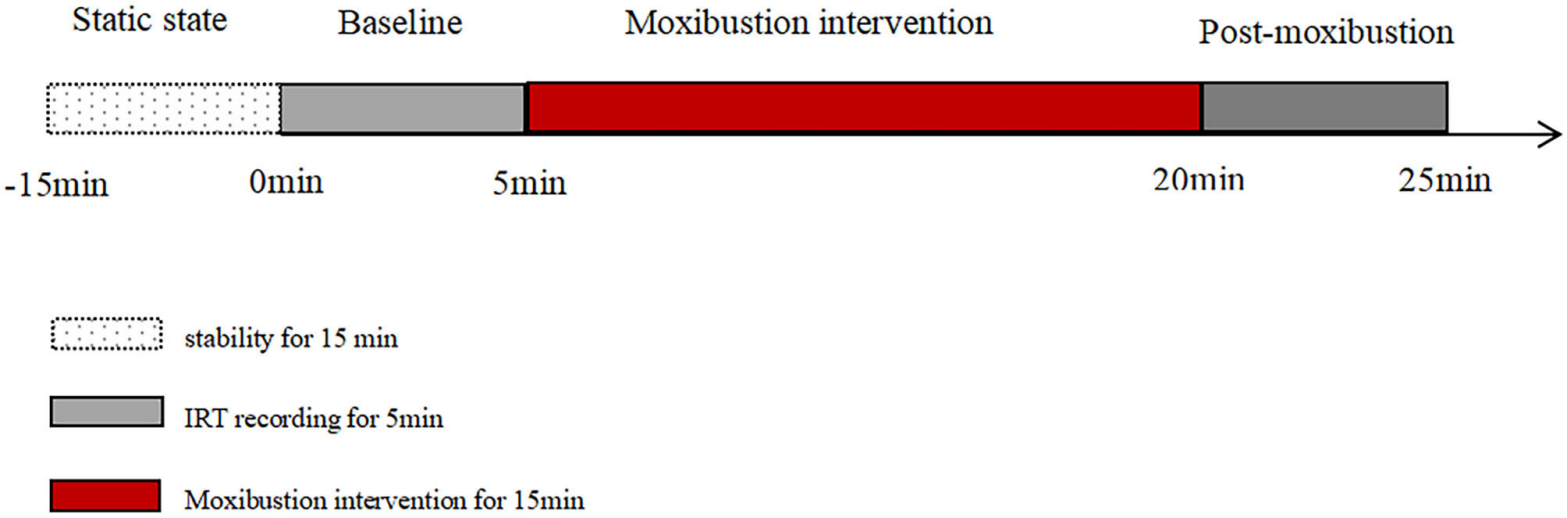
Procedures for the IRT examination.

The positions of the 6 measuring sites in the left forearm are shown in Figure 2 as follows: Site 1 (Chize, LU5), Site 2 (midpoint of LU9 and LU5), Site 3 (Taiyuan, LU9), Site 4 (Shaohai, HT3), Site 5 (midpoint of HT7 and HT3), and Site 6 (Shenmen, HT7). As shown in Figure 3, the total experimental duration for the two groups was 40 min, including a 15-min rest period, 5-min baseline assessment, 15-min moxibustion intervention period, and 5-min postintervention period.

The participants were asked to remain still for 15 min in a supine position to adapt to the room temperature prior to the IRT examination. After 15 min of inactivity, the researcher positioned the test sites of the heart and lung meridians in the left forearm. A researcher placed the blunt head of a cotton swab over the six measuring sites to help another researcher locate these sites in the screen of the thermograph. Using the automatic interval saving shooting mode, one thermal picture was taken every minute. In this study, suspended moxibustion was performed by holding an ignited moxa stick at a certain distance (about 3–4 cm) over the skin surface, keeping the moxibustion site warm without burning the skin. In the heart meridian intervention group, moxibustion was applied over Site 4 (HT3) of the heart meridian (Figure 1B). In the lung meridian intervention group, moxibustion was performed over Site 1 (LU5) of the lung meridian (Figure 1A). During moxibustion, the temperatures of the heart and lung meridians were measured.

### Analysis of IRT

The temperatures of the measurement sites along the lung and heart meridians on the left forearm were analyzed using the software NS9500Std (Avio Infrared Technologies Co., Ltd., Tokyo), including LU9, the midpoint of LU9 and LU5, LU5, HT7, the midpoint of HT7 and HT3. The temperature change from pre-moxibustion (ΔT) at 5/10/15 min after moxibustion and 5-min post-moxibustion at these measurement sites was also analyzed.

### Statistical Analysis

SPSS 22.0 (SPSS Inc., Chicago, IL, USA) was used to perform statistical analysis. The numerical data with a normal distribution are expressed as the mean ± standard error of the mean (SEM), whereas the data with a skewed distribution are expressed as the median and interquartile ranges.

The paired *t*-test was used to compare differences in fundamental physiological parameters between before and immediately after the IRT examination. Temperature change over time within and between different measurement sites were analyzed using repeated measures analysis of variance (ANOVA). If significant differences were detected, Bonferroni *post-hoc* multiple comparison test was performed. A *p*-value of < 0.05 was considered statistically significant.

## Results

### Demographics Characteristics of Participants

One hundred and two participants were screened, and 80 eligible subjects were finally included. Among them, 40 participants (aged 28.08 ± 0.54 years) were assigned to the lung meridian intervention group, and 40 participants (aged 27.90 ± 0.52 years) were assigned to the heart meridian intervention group. All 80 participants completed the trial. The demographics characteristics of participants in both groups are shown in Table 1. There were no significant differences in gender, age, height, weight, and Body Mass Index (BMI) of participants between lung meridian intervention group and heart meridian intervention group (*P* > 0.05).

**Table 1:** Demography characteristics of participants in experiments (mean ± SEM).

**Demographic characteristics**	**Group**	
	**Lung meridian intervention group (*n* = 40, 20 male/20 female)**	**Heart meridian intervention group (*n* = 40, 20 male/20 female)**
Age (YEARS)	28.08 ± 0.54	27.90 ± 0.52
Height (CM)	167.90 ± 1.46	168.85 ± 1.48
Weight (KG)	62.62 ± 2.52	62.86 ± 2.65
BMI (Kg/m^2^)	21.93 ± 0.56	21.79 ± 0.65

*mean ± SEM, mean ± standard error of mean; BMI, body mass index*.

### Comparison of Physiological Parameters Before and Immediately After IRT

The data shown in Table 2 suggest that there were no statistically significant differences in systolic blood pressure, diastolic blood pressure, heart rate, respiratory rate or body temperature immediately after IRT did not significantly differ compared with baseline in both groups (*P* > 0.05), suggesting that a relatively stable physiological state was maintained during the IRT examination, thereby confirming that the changes in the heart and lung meridian temperatures were not influenced by significant changes in physiological activity.

**Table 2:** Physiological indexes of the study subjects prior to and immediately after IRT examination (mean ± SEM, *n* = 80).

**Physiological indexes**	**Lung meridian intervention group**				**Heart meridian intervention group**			
	**Prior to IRT examination**	**Immediately after IRT examination**	***Z***	***P***	**Prior to IRT examination**	**Immediately after IRT examination**	***Z***	***P***
SBP (MMHG)	110.88 ± 2.02	109.98 ± 2.05	0.72	0.47	111.53 ± 2.23	110.13 ± 2.07	0.56	0.57
DBP (MMHG)	72.68 ± 1.52	72.40 ± 1.33	0.11	0.91	72.40 ± 1.49	72.53 ± 1.25	0.15	0.88
HR (BPM)	71.63 ± 1.58	69.70 ± 1.39	1.23	0.20	71.58 ± 1.58	69.98 ± 1.06	1.51	0.13
RR (RPM)	16.05 ± 0.24	16.10 ± 0.23	0.19	0.85	16.18 ± 0.25	16.10 ± 0.24	0.14	0.89
BT (°C)	36.28 ± 0.05	36.32 ± 0.04	0.73	0.47	36.35 ± 0.04	36.36 ± 0.04	0.07	0.95

*SBP, systolic blood pressure; DBP, diastolic blood pressure; HR, heart rate; RR, respiratory rate; BT, body temperature; mmHg, millimeters of mercury; bpm, beats per minute; rpm, breaths per minute*.

### Temperature and Its Corresponding Magnitude of Change in Both Meridians

#### The Lung Meridian Intervention Group

**Figure 4:**
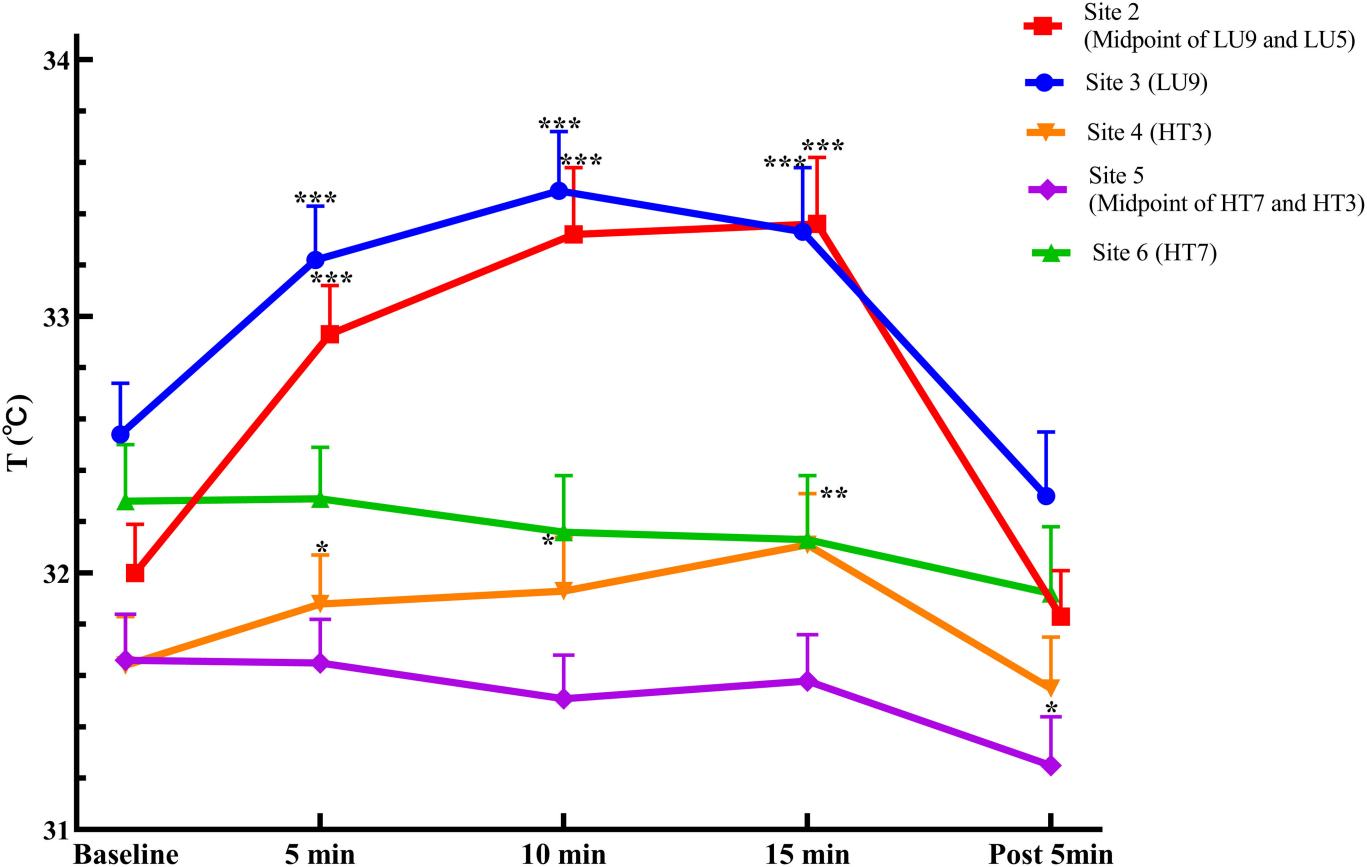
Temperature curves in measurement sites of both meridians in the Lung intervention group (mean ± SEM, °C). ***P < 0.001, **P < 0.01, and *P < 0.05, compared with pre-moxibustion.

The temperatures of the corresponding measurement sites in both meridians in the lung meridian intervention group are summarized in Table 3; Figure 4. Following moxibustion over Site 1 (LU5) of the lung meridian, the temperature of the distal sites in the lung meridian (i.e., Site 2 and Site 3) increased significantly at 5, 10, and 15 min after moxibustion compared with pre-moxibustion (*P* < 0.001). After the termination of moxibustion, the temperature of Site 1 and Site 2 decreased slightly compared with pre-moxibustion, but the change was not statistically significant. On the other hand, compared to the lung meridian, the temperature of Site 4 in the heart meridian, which was nearest to the moxibustion site, increased significantly at 5, 10, and 15 min after moxibustion compared with pre-moxibustion (*P* < 0.05), while the temperature in the distal sites of the heart meridian (i.e., Site 5 and Site 6) did not change significantly during moxibustion (*P* > 0.05).

**Table 3:** Temperature and its corresponding change magnitude in measurement Sites of both meridians in the Lung intervention group (mean ± SEM, °C).

**Measurement sites**	**Pre-moxibustion**	**During moxibustion**			**Post-moxibustion 5 min**
		**Moxibustion 5 min**	**Moxibustion 10 min**	**Moxibustion 15 min**	
Site 1 (LU5)	32.31 ± 0.22 —	42.36 ± 0.73*** (10.04 ± 0.80)^a3b3c3^	42.26 ± 0.51*** (9.95 ± 0.55)^a3b3c3^	40.97 ± 0.43*** (8.66 ± 0.49)^a3b3c3^	33.91 ± 0.13*** (1.60± 0.18)^a3b3c3^
Site 2 (Midpoint of LU9 and LU5)	32.00 ± 0.19 —	32.93 ± 0.19*** (0.93 ± 0.14)^a1, b3, c3^	33.32 ± 0.26*** (1.32 ± 0.23)^a1, b3, c3^	33.36 ± 0.26*** (1.35 ± 0.26)^b3, c3^	31.83 ± 0.18 (−0.17 ± 0.11)
Site 3 (LU9)	32.54 ± 0.20 —	33.22 ± 0.21*** (0.68 ± 0.12)^b2, c1^	33.49 ± 0.23*** (0.95 ± 0.15)^b3, c3^	33.33 ± 0.25*** (0.79 ± 0.15)^b2, c2^	32.30 ± 0.25 (−0.24 ± 0.12)
Site 4 (HT3)	31.64 ± 0.19 —	31.88 ± 0.19* (0.24 ± 0.07)	31.93 ± 0.21* (0.29 ± 0.09)	32.11 ± 0.20** (0.47 ± 0.10)^b1, c1^	31.55 ± 0.20 (−0.09 ± 0.11)
Site 5 (Midpoint of HT7 and HT3)	31.66 ± 0.18	31.65 ± 0.17 (-0.01 ± 0.09)	31.51 ± 0.17 (−0.15 ± 0.11)	31.58 ± 0.18 (−0.08 ± 0.13)	31.25 ± 0.19* (−0.41 ± 0.12)
Site 6 (HT7)	32.28 ± 0.22 —	32.29 ± 0.20 (0.01 ± 0.13)	32.16 ± 0.22 (−0.12 ± 0.15)	32.13 ± 0.25 (−0.15 ± 0.19)	31.92 ± 0.26 (−0.36 ± 0.18)

The values in parentheses were the increased (or decreased) change magnitude of temperature from pre-moxibustion; adjusted P-value based on the Bonferroni post-hoc multiple comparison test; ***p < 0.001, **p < 0.01, and *p < 0.05 compared with pre-moxibustion; ^a3^p < 0.001, and ^a1^p < 0.05 compared with Site 4; ^b3^p < 0.001, ^b2^p < 0.01, and ^b1^p < 0.05 compared with Site 5; ^c3^p < 0.001, ^c2^p < 0.01, and ^c1^p < 0.05 compared with Site 6.

Regarding the comparison of temperature change magnitude from baseline (ΔT) between the two meridians, following moxibustion over LU5 of the lung meridian, the ΔT of Site 2 in the lung meridian was significantly higher than Site 4 in the heart meridian at 5 and 10 min after moxibustion (*P* < 0.05), despite that Site 2 was more distal from the moxibustion site than Site 4. Similarly, the ΔT of Site 3 in the lung meridian was significantly higher than Site 5 and Site 6 in the heart meridian at 5, 10 and 15 min after moxibustion (*P* < 0.05), despite that the Site 3 was more distal from the moxibustion site than Site 5 and Site 6.

#### The Heart Meridian Intervention Group

**Figure 5:**
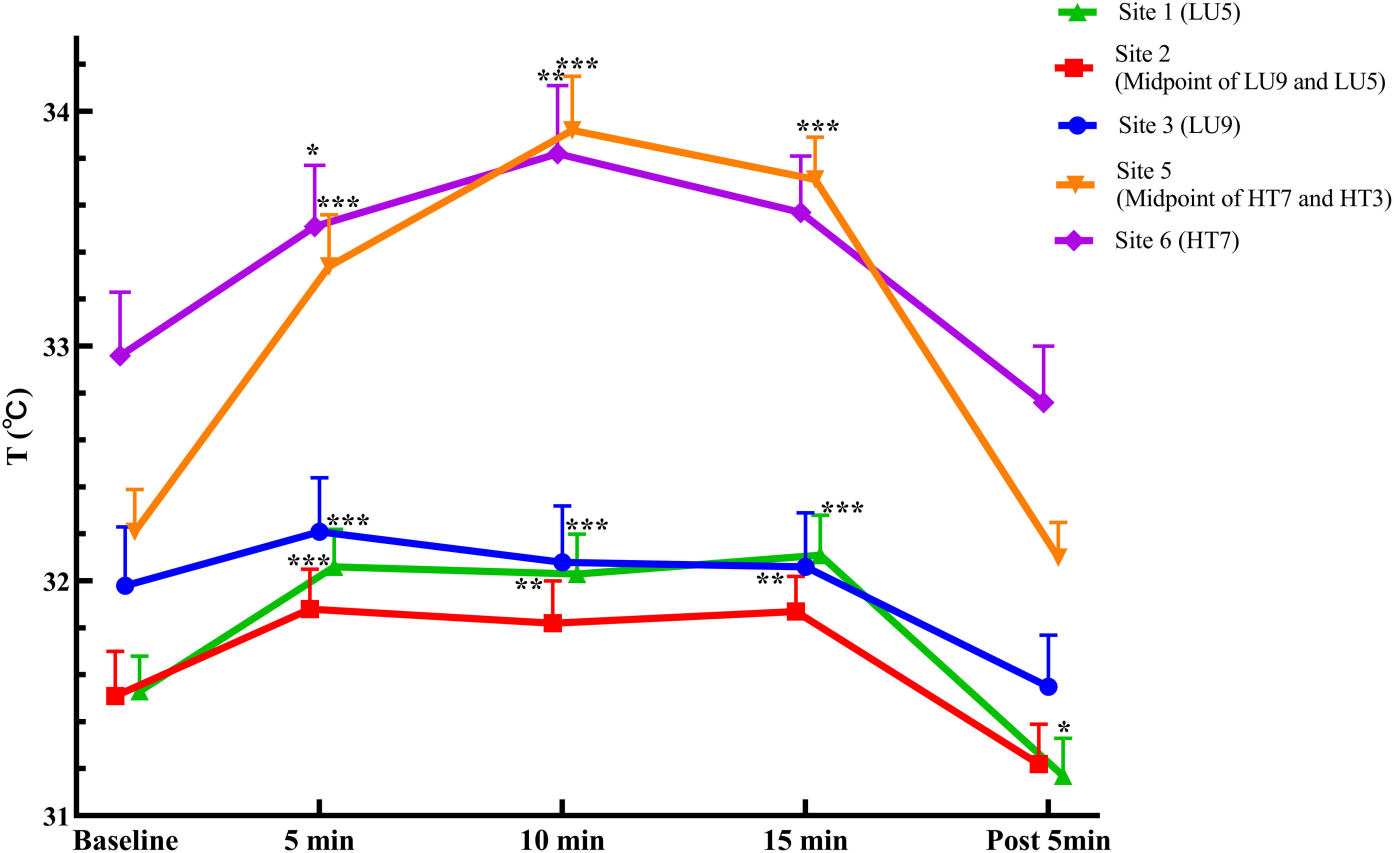
Temperature curves in measurement sites of both meridians in the Heart intervention group (mean ± SEM, °C). ***P < 0.001, **P < 0.01, and *P < 0.05, compared with pre-moxibustion.

The temperatures of the corresponding measurement sites along both meridians in the heart meridian intervention group are summarized in Table 4; Figure 5. Following moxibustion over Site 4 (HT3) of the heart meridian, the temperature of the distal sites in the heart meridian (i.e., Site 5) increased significantly at 5, 10, and 15 min after moxibustion compared with pre-moxibustion (*P* < 0.001). The temperature of the most distal site in the heart meridian (i.e., Site 6) increased significantly at 5, 10 min after moxibustion compared with pre-moxibustion (*P* < 0.05). After the termination of moxibustion, the temperature of Site 5 and Site 6 decreased slightly compared with pre-moxibustion, but the change was not statistically significant (*P* > 0.05).

**Table 4:** Temperature and its corresponding change magnitude in measurement Sites of both meridians in the Heart intervention group (mean ± SEM, °C).

**Measurement sites**	**Pre-moxibustion**	**During moxibustion**			**Post-moxibustion 5 min**
		**Moxibustion 5 min**	**Moxibustion 10 min**	**Moxibustion 15 min**	
Site 1 (LU5)	31.53 ± 0.15 —	32.06 ± 0.16*** (0.54 ± 0.09)	32.03 ± 0.17*** (0.49 ± 0.10)	32.11 ± 0.17*** (0.58 ± 0.12)	31.17 ± 0.16* (−0.36 ± 0.11)
Site 2 (Midpoint of LU9 and LU5)	31.51 ± 0.19 —	31.88 ± 0.17*** (0.38 ± 0.08)	31.82 ± 0.18** (0.32 ± 0.07)	31.87 ± 0.15** (0.36 ± 0.08)	31.22 ± 0.17 (−0.29 ± 0.11)
Site 3 (LU9)	31.98 ± 0.25 —	32.21 ± 0.23 (0.23 ± 0.10)	32.08 ± 0.24 (0.11 ± 0.12)	32.06 ± 0.23 (0.08 ± 0.16)	31.55 ± 0.22 (−0.43 ± 0.17)
Site 4 (HT3)	32.14 ± 0.19 —	42.08 ± 0.79*** (9.94 ± 0.77)^a3, b3, c3^	41.02 ± 0.49*** (8.88 ± 0.54)^a3, b3, c3^	43.44 ± 2.29*** (11.29 ± 2.29)^a3, b3, c3^	33.83 ± 0.14*** (1.68 ± 0.19)^a3, b3, c3^
Site 5 (Midpoint of HT7 and HT3)	32.21 ± 0.18	33.34 ± 0.22*** (1.12 ± 0.19)^c1^	33.92 ± 0.23*** (1.71 ± 0.21)^a2, b3, c3^	33.71 ± 0.18*** (1.49 ± 0.20)^b2, c3^	32.10 ± 0.15 (−0.12 ± 0.14)
Site 6 (HT7)	32.96 ± 0.27 —	33.51 ± 0.26* (0.55 ± 0.17)	33.82 ± 0.29** (0.86 ± 0.21)	33.57 ± 0.24 (0.61 ± 0.21)	32.76 ± 0.24 (−0.20 ± 0.17)

The values in parentheses were the increased (or decreased) change magnitude of temperature from pre-moxibustion; adjusted P-value based on the Bonferroni post-hoc multiple comparison test; ***p < 0.001, **p < 0.01 and *p < 0.05 compared with pre-moxibustion; ^a3^p < 0.001 and ^a2^p < 0.01 compared with Site 4; ^b3^p < 0.001 and ^b2^p < 0.01 compared with Site 5; ^c3^p < 0.001 and ^c1^p < 0.05 compared with Site 6.

Compared with the heart meridian, the temperature of Site 1 in the lung meridian, which was nearest to the moxibustion site, increased significantly at 5, 10, or 15 min after moxibustion and 5 min following the removal of moxibustion (*P* < 0.05). The temperature at the distal site of the lung meridian (i.e., Site 2) increased significantly at 5, 10, or 15 min after moxibustion (*P* > 0.05), while the temperature at the most distal site of the lung meridian (i.e., Site 3) did not change significantly at all measurement timepoints during moxibustion and post-moxibustion phases (*P* > 0.05).

Moreover, regarding the comparison of temperature change magnitude from baseline (ΔT) between the two meridians, following moxibustion over HT3 of the heart meridian, the ΔT of Site 5 in the heart meridian was significantly higher than Site 1 of the lung meridian at 10 min after moxibustion (*P* < 0.05), despite that Site 5 was more distal from the moxibustion site than Site 1.

#### Representative Infrared Images

**Figure 6:**
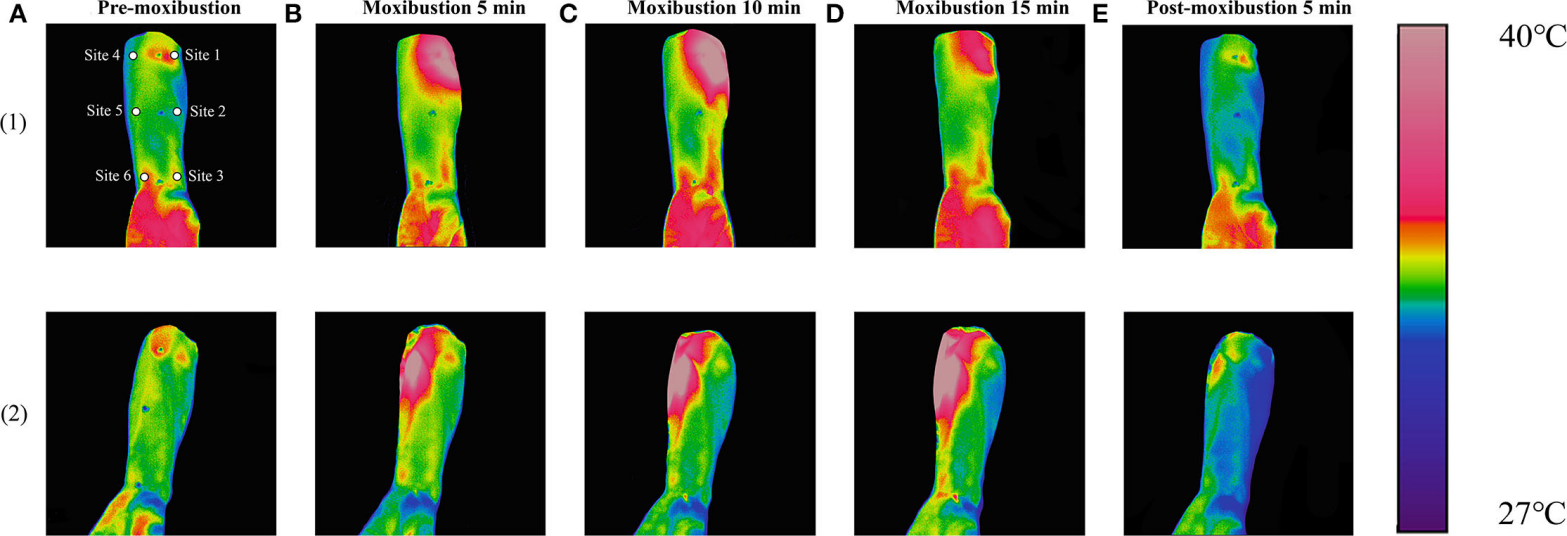
Continuous display of representative infrared images. **(1)** Infrared images in the lung intervention group and **(2)** Infrared images in the heart intervention group. **(A)** images with pre-moxibustion, **(B)** images with moxibustion 5 min, **(C)** images with moxibustion 10 min, **(D)** images with moxibustion 15 min, and **(E)** Images with post-moxibustion 5 min.

Infrared images of the representative subject are shown in Figure 6. As shown in Figure 6(1), following moxibustion over Site 1 (LU5) of the lung meridian, the temperature of the local and distal sites in the lung meridian increased significantly, presenting as a high-temperature red stripe along the radial side of the forearm that was basically coincided with the route of the lung meridian in the forearm, while no high-temperature red stripe appeared in the route of the heart meridian in the forearm (ulnar side of the forearm).

As shown in Figure 6(2), following moxibustion over Site 4 (HT3) of the heart meridian, the temperature of the local and distal sites in the heart meridian increased significantly, presenting as a high-temperature red stripe along the ulnar side of the forearm that was basically coincided with the route of the heart meridian in the forearm, while no high-temperature red stripe appeared in the route of the lung meridian in the forearm (radial side of the forearm).

## Discussion

Temperatures of acupuncture points and meridians can reflect the physiological and pathological state related to the function of meridians (25). There is a correlation between skin-specific temperatures and the structure of meridians; for example, it has been demonstrated that temperatures of meridian routes and acupuncture points are higher than those of non-meridian routes and non-acupuncture points (26, 27). Given that it can sensitively detect infrared radiation and temperature on the body surface, the interest in the application of IRT has increased among meridian researchers in recent years (28, 29). However, the vast majority of previous studies have focused on one meridian and its corresponding organ to investigate the somato-viscera correlation, or the differences between meridians (or acupuncture points) and non-meridians (or non-acupuncture points). To date, this is the first study that was designed to explore the specificity of site-to-site associations on the body surface between different meridians by using IRT.

### Principal Findings

In the lung meridian intervention group, regarding the comparison of temperature change magnitude from baseline (ΔT) between the two meridians, the ΔT of Site 2 in the lung meridian was significantly higher than Site 4 in the heart meridian at 5 and 10 min after moxibustion (*P* < 0.05), despite that Site 2 was more distal from the moxibustion site than Site 4. Similarly, the ΔT of Site 3 in the lung meridian was significantly higher than Site 5 and Site 6 in the heart meridian at 5, 10, and 15 min after moxibustion (*P* < 0.05), despite that the Site 3 was more distal from the moxibustion site than Site 5 and Site 6. On the other hand, in the heart meridian inverention group, when moxibustion was performed over HT3 of the heart meridian, similar response was observed but in the heart meridian. The thermal transport effect of the distal sites along the heart meridian was more significant than that of the site closer to the moxibustion site but located in the lung meridian. These results indicate that in the heart and lung meridians, the moxibustion-induced thermal transport effect is closely related to meridian routes, not just related to the absolute distance from the moxibustion site, thereby confirming the relative specificity of “site-to-site” associations on the body surface in these two meridians.

Regarding possible factors and mechanisms that cause the difference in the thermal transport effect between these two meridians. First, from the perspective of anatomy, previous studies have indicated that the physical structure of meridians have close correlation with blood vessels, nerves, lymphatic vessels, and neurovascular bundles (30–33). These anatomical structures are usually distributed longitudinally in the forearm. Meridians are likely to be three-dimensional channels of multiple structures that transmit substance, heat, and energy, thereby the thermal transport effect along the meridian direction is more significant than that along the nonmeridian direction. Second, previous studies by our team yielded similar results using other techniques (i.e., laser Doppler flowmeter), which demonstrated that the change of blood flow induced by moxibustion was generally more significant in the distal sites along the corresponding meridian than that in the closer sites of the other meridian (34). The level of skin blood flow is subject to both reflex thermoregulatory control and influences from the direct effects of skin temperature (35). Previous studies have observed a nonlinear relationship between blood flow and skin temperature in the forearm, which involves a process of “initial vessel expansion-peak-return to baseline” (36). In addition, the effect of changes in skin temperature may be caused by a combination of mechanisms involving endothelial, adrenergic, and sensory systems (37). Theoretically, the aforementioned factors and mechanisms may partly explain the principle findings of our study. Nonetheless, since this study mainly focuses on the biological phenomena of the meridians, the relevant underlying mechanisms will be further explored in our future research.

In addition, the results of the present study indicate that there is no significant difference between heart and lung meridian temperatures in healthy human subjects before moxibustion interventions, suggesting that the meridian system of a healthy body is generally in a state of balance, which is consistent with the results of previous studies (38, 39). Our study revealed that acupuncture points are in a dynamic state; that is, the acupuncture points of a healthy body are in a resting state and no significant differences exist between acupuncture points and non-acupuncture points, while acupuncture points turn “active” under interventions such as moxibustion.

### Highlights and Strengths of the Present Study

First, the moxibustion-induced thermal effects on the same cross-section of two different meridians were explored. Given that the lung and heart meridians have relatively short distribution routes, similar distribution locations and relatively independent functions, these two meridians were chosen for comparison in this study. To the best of our knowledge, this is the first IRT study to investigate the specificity of the site-to-site association between two meridians by comparing the moxibustion-induced thermal changes between the heart and lung meridians.

Second, the influence of confounding factors on the results was minimized. On the one hand, the accuracy of IRT in measuring the meridian temperature is affected by manual repetitive localization errors. Therefore, we used a cotton swab-assisted localization method. Remarkably, this novel method could achieve accurate positioning of temperature measurement sites without affecting the skin temperature, so it does not affect the experimental results. Nevertheless, body surface temperature is influenced by physiological, pathological, and environmental factors. The physiological factors mainly include the skin color (40), circadian rhythm (41), sex (42, 43), menstrual cycle, subcutaneous fat and metabolic rate. The pathological factors mainly involve specific disease states. The environmental factors mainly include the room size (44), ambient temperature (45), and relative humidity (46). Therefore, the subjects in this study were selected strictly based on the inclusion and exclusion criteria, and the subjects were all age-matched with a gender ratio of 1:1 in the two groups. Moreover, in this study, the room temperature and humidity were controlled, and the subjects were provided sufficient time to adapt to the environment. In addition, the fundamental physiological parameters of all subjects were measured before and after IRT, allowing us to determine whether the changes in body surface temperature were due to physiological changes. All these experimental designs greatly reduce the inaccuracy of the data and ensure the credibility of the findings.

### Limitations

The limitation of this study is that IRT, as well as human body temperature, are influenced by many factors. Although multiple measures were taken to control some of these factors as much as possible during our experiments, we cannot rule out the interference of other unknown factors. In addition, given that only two meridians and limited measurement sites were compared using IRT, all current findings are not sufficiently robust. Last but not the least, with the aim to reduce individual difference as much as possible between two groups of different participants, the group design of our study would have been more reasonable and feasible if another group of the same participants were added to compared the thermal transport effect of the heart and lung meridians, which will be further investigated in the future study.

## Conclusions

In the heart and lung meridians, the moxibustion-induced thermal transport effect is closely related to meridian routes, not just related to the absolute distance from the moxibustion site, thereby confirming the relative specificity of “site-to-site” connections on the body surface in these two meridians.

## Data Availability Statement

The original contributions presented in the study are included in the article/supplementary files, further inquiries can be directed to the corresponding author/s.

## Ethics Statement

The studies involving human participants were reviewed and approved by the Third Affiliated Hospital of Zhejiang Chinese Medical University, Hangzhou City, Zhejiang Province, China. The patients/participants provided their written informed consent to participate in this study. Written informed consent was obtained from the individual(s) for the publication of any potentially identifiable images or data included in this article.

## Author Contributions

XL performed the data analysis and wrote the manuscript. JiF, YW, YZ, and JuF designed the experiments. YJ, XH, and HH scrubbed the data and maintained the research data. YW, YZ, and JuF checked the data. XS conceived and designed the experiments. All authors contributed to the article and approved the submitted version.

## Funding

The trial is financially supported by the National Key Research and Development Program of China (No. 2018YFC1704600) and the Postgraduate Scientific Research Fund of Zhejiang Chinese Medical University (No. 2020YKJ04).

## Conflict of Interest

The authors declare that the research was conducted in the absence of any commercial or financial relationships that could be construed as a potential conflict of interest.

## Publisher's Note

All claims expressed in this article are solely those of the authors and do not necessarily represent those of their affiliated organizations, or those of the publisher, the editors and the reviewers. Any product that may be evaluated in this article, or claim that may be made by its manufacturer, is not guaranteed or endorsed by the publisher.
